# Urocortin-2 Prevents Dysregulation of Ca^2+^ Homeostasis and Improves Early Cardiac Remodeling After Ischemia and Reperfusion

**DOI:** 10.3389/fphys.2018.00813

**Published:** 2018-07-03

**Authors:** Alejandro Domínguez-Rodríguez, Isabel Mayoral-Gonzalez, Javier Avila-Medina, Eva S. de Rojas-de Pedro, Eva Calderón-Sánchez, Ignacio Díaz, Abdelkrim Hmadcha, Antonio Castellano, Juan A. Rosado, Jean-Pierre Benitah, Ana M. Gomez, Antonio Ordoñez, Tarik Smani

**Affiliations:** ^1^Cardiovascular Pathophysiology, Institute of Biomedicine of Seville, University Hospital of Virgen del Rocío, University of Seville, CIBERCV, CSIC, Seville, Spain; ^2^Departamento de Fisiología Médica y Biofísica, Universidad de Sevilla, Seville, Spain; ^3^Department of Regeneration and Cell Therapy, Andalusian Center for Molecular Biology and Regenerative Medicine (CABIMER), Junta de Andalucia, University of Pablo de Olavide, University of Seville, CSIC, Seville, Spain; ^4^Centro de Investigación Biomédica en Red de Diabetes y Enfermedades Metabólicas Asociadas, Madrid, Spain; ^5^Departamento de Fisiología, Universidad de Extremadura, Cáceres, Spain; ^6^UMR-S 1180, INSERM, Universite Paris-Sud, Université Paris-Saclay, Châtenay-Malabry, France

**Keywords:** Urocortin-2, ischemia and reperfusion, adverse remodeling, Ca^2+^ dysregulation, store operated Ca^2+^ channels

## Abstract

**Aims:** Urocortin-2 (Ucn-2) is a potent cardioprotector against Ischemia and Reperfusion (I/R) injuries. However, little is known about its role in the regulation of intracellular Ca^2+^ concentration ([Ca^2+^]_i_) under I/R. Here, we examined whether the addition of Ucn-2 in reperfusion promotes cardioprotection focusing on ([Ca^2+^]_i_ handling.

**Methods and Results:** Cardiac Wistar rat model of I/R was induced by transient ligation of the left coronary artery and experiments were conducted 1 week after surgery in tissue and adult cardiomyocytes isolated from risk and remote zones. We observed that I/R promoted significant alteration in cardiac contractility as well as an increase in hypertrophy and fibrosis in both zones. The study of confocal [Ca^2+^]_i_ imaging in adult cardiomyocytes revealed that I/R decreased the amplitude of [Ca^2+^]_i_ transient and cardiomyocytes contraction in risk and remote zones. Interestingly, intravenous infusion of Ucn-2 before heart’s reperfusion recovered significantly cardiac contractility and prevented fibrosis, but it didn’t affect cardiac hypertrophy. Moreover, Ucn-2 recovered the amplitude of [Ca^2+^]_i_ transient and modulated the expression of several proteins related to [Ca^2+^]_i_ homeostasis, such as TRPC5 and Orai1 channels. Using Neonatal Rat Ventricular Myocytes (NRVM) we demonstrated that Ucn-2 blunted I/R-induced Store Operated Ca^2+^ Entry (SOCE), decreased the expression of TRPC5 and Orai1 as well as their interaction in reperfusion.

**Conclusion:** Our study provides the first evidences demonstrating that Ucn-2 addition at the onset of reperfusion attenuates I/R-induced adverse cardiac remodeling, involving the [Ca^2+^]_i_ handling and inhibiting the expression and interaction between TRPC5 and Orai1.

## Introduction

Ischemic heart disease still remains the leading cause of death worldwide, and STEMI is the most common type of heart infarcts (World Health Organization, 2008). The majority of patients with acute myocardial infarction are routinely treated with pharmacological reperfusion therapy and/or widening the vessel with angioplasty (Ribichini and Wijns, 2002). Actually, an effective and early revascularization limits the extent of myocardial necrosis and left ventricular dysfunction. However, critical injuries occur when oxygen rich blood reperfuses the vulnerable myocardial tissue, notably by extensive production of the mitochondrial reactive oxygen species. This phenomenon is known as I/R syndrome (Hausenloy et al., 2017). I/R injuries usually lead to adverse cardiac remodeling and further development of heart failure. The initial myocardial responses related to the early adverse cardiac remodeling involve the loss of cardiomyocytes, the hypertrophy of remaining cardiomyocytes as well as changes in composition and distribution of components of the extracellular matrix, especially the generation of collagen and fibrosis extension to the not-infarcted myocardial remote area (Pfeffer and Braunwald, 1990). Generally, heart failure is characterized at the molecular level by dysfunction and abnormalities of the handling of [Ca^2+^]_i_ with significant alteration in excitation-contraction coupling (Gómez et al., 1997; Lehnart et al., 2009). Previous reports have shown that cardiac dysfunction observed in patients with heart failure was related to altered expression and activity of Ca^2+^-related proteins (Kho et al., 2012). More recently, compelling evidences have demonstrated that different cationic channels which permeate Ca^2+^, such as TRPC and store operated Ca^2+^ channels (SOCC) contribute to cardiomyopathies, cardiac fibrosis and cardiac remodeling (Smani et al., 2015).

In the last decade, Urocortin peptides (Ucn-1, Ucn-2, Ucn-3) belonging to the CRF family (Dautzenberg and Hauger, 2002), have emerged as a potential therapeutic agonists that improved heart performances and protected it from I/R injuries (Boonprasert et al., 2008). In heart, Ucn peptides bind to CRF-R2 receptor enhancing cAMP production (Brar et al., 1999) and Protein Kinase A (PKA) activation (Calderón-Sanchez et al., 2009). We have demonstrated recently that Ucn-1 activated the other important effector of cAMP, Epac (Calderón-Sánchez et al., 2016). Epac and ERK1/2 are also involved in the Ucn-1-induced positive inotropism, in its regulation of apoptotic genes and in microRNAs expression during heart reperfusion (Díaz et al., 2017). A recent study has demonstrated that sustained activation of Epac activated Ca^2+^ influx through store-operated Ca^2+^ entry (SOCE) via TRPC3/4 channels (Domínguez-Rodríguez et al., 2015). Here, we analyzed the potential protective effects of the infusion of Ucn-2 in rat model of I/R before reperfusion, focusing on its effect in [Ca^2+^]_i_ handling and its regulation of Ca^2+^-related key proteins during adverse cardiac remodeling. We demonstrate that Ucn-2 recovers significantly hemodynamic parameters of heart subjected to I/R, prevents fibrosis and decreases infarct size. Ucn-2 also improves the handling of [Ca^2+^]_i_ abnormalities caused by I/R and attenuates SOCE through downregulation of TRPC5 and Orai1.

## Materials and Methods

All the experiments with animals were performed in accordance with the recommendations of the Royal Decree 53/2013 in agreement to the Directive 2010/63/EU of the European Parliament and approved by the local Ethics Committee on human Research of the “Virgen del Rocio” University Hospital of Seville and the Animal Research Committee of the University of Seville.

### Induction of Rat Myocardial Infarction and Reperfusion (I/R)

Male Wistar rats weighing 250 ± 50 g were anesthetized with a mixture of O_2_/sevoflurane 2%, then with anesthesia (50 mg/kg ketamine plus 8 mg/kg xylazine i.p) as described previously (Díaz et al., 2017). A small animal ventilator was used (Harvard Apparatus, Holliston, MA, United States) with a tidal volume (V_t_) of 1.5–2.0 ml and 75–80 ventilations per minute. A left thoracotomy was performed in the intercostal space between the third and fourth ribs, followed by a pericardiotomy. The LCA was occluded with a 6-0 ProleneTM silk suture (Ethicon, Spain) and tied off below the level of the left atria appendage and over a small tube placed into the suture for the posterior release of the occlusion, for a correct reperfusion. LCA occlusion was confirmed by visual observation of cyanosis and ST-segment elevation by continuous ECG monitoring. After 40 min of LCA ligation, reperfusion was initiated by releasing the knot, removing the tube and was confirmed by the appearance of epicardial hyperemic and by ECG recovery. The chest cavity was closed and the air was expelled from the chest, sevoflurane administration was switched off and the animal was supplied with O_2_ until reflexes were detected. Analgesia was induced with meloxicam (1 mg/kg) administered subcutaneously. Rats were left on a heating pad until fully conscious recovery.

For this study, we considered the following experimental groups: Group 1 (I/R): ischemia was produced by LCA ligation during 40 min and the administration of vehicle (saline 0.9% NaCl) by tail vein injection 5 min before reperfusion. Group 2 (I/R + Ucn-2): ischemia was produced by LCA ligation during 40 min and an intravenous (*i.v*.) dose of Ucn-2 (150 μg/Kg) was administered by tail vein injection 5 min before reperfusion. Group 3 (Sham): operated rats underwent the same surgical procedure without coronary ligation. The same volume of vehicle (saline 0,9% NaCl) was administrated by tail vein injection.

Animals were randomly subjected to either LCA ligation or sham operation (5% mortality during the interventions). The survival rate in all groups after Ucn-2 or saline treatment was 95–100%.

### Echocardiography

Transthoracic echocardiographic analyses were performed using Vevo^TM^ 2100 ultrasound system with transducer MS250 with a frequency range of 13-24 MHz (VisualSonics^TM^, Toronto, ON, Canada). The cardiac function was analyzed after surgery in light anesthetized rat with 2% sevoflurane. Breath and heart rate as well as rectal core temperature were monitored. A water heater and a heat lamp were used to keep the temperature around 37°C. M-Mode images of the left ventricle at the level of the papillary muscles were obtained, and different functional hemodynamic parameters were evaluated. Images acquisition and analyses were done in a blind manner.

### Cardiovascular Magnetic Resonance

The cardiovascular magnetic resonance study was performed with the imaging system ICON 1T (Bruker, Rheinstetten, Germany) using a rat whole body coil. Animals were sedated by sevoflurane (2–4%) and monitored by electrocardiogram and rectal thermometer, checking the maintenance of appropriate physiological and hemodynamic conditions (heart rate between 400 and 500 beats per minute, respiratory rate 60–100 breaths per minute and body temperature > 35°C). To evaluate heart function, images were collected with a T1-weighted spin echo cine sequences and synchronized with the ECG (repetition time: 15 ms, echo time 2 ms, resolution: 0.234 × 0.234 mm, slice thickness: 1.25 mm, 6–7 consecutive cuts without spacing between them) in short-axis plane from baseline to the height of the mitral valve to the left ventricular apex. Subsequently, to quantify the ischemic area, images were collected with gradient echo T1 sequences and synchronized with the ECG (repetition time: 100 ms, echo time: 2.5 ms, resolution: 0.234 × 0.234 mm, slice thickness: 1.250 mm, angle of rotation: 75° or 90°, 2 cuts with the same geometry as the previous film sequences) to approximately 15 min after the introduction of a gadolinium-based contrast which highlights fibrotic areas.

### Infarct Size Determination

After intraperitoneal injection of pentobarbital sodium (100 mg/Kg), cardiectomy was done 1 week after surgical intervention. Hearts were immersed in cold 1x PBS to be clean of blood. Then, they were frozen during 1 h and a half at -20°C and sliced in 2 mm. The slices were immersed in a solution of 1% Triphenyltetrazolium Chloride (TTC) in PBS at 37°C during 30 min. The healthy myocardium shows an intense red color because of the redox activity of dehydrogenases; meanwhile the necrosed area remains white. The heart slices were photographed and the areas of infarction were quantified using ImageJ (NIH, United States). The infarct size was measured as a percentage of total left ventricle at the level of papillary muscles.

### Cardiomyocyte Area

Wheat Germ Agglutinin (WGA) staining was used to assess cardiomyocyte cross-sectional-area as previously described (Söderberg et al., 2008; Bensley et al., 2016). Paraffin heart sections (6 μm) were de-waxed and rehydrated 3 times with xylene and further washed with ethanol. Then, they were washed 2 min in running water before the staining. Heart sections were stained with WGA-Alexa Fluor 488 (Life technologies, United States) at 10 μg/ml as well as DAPI (Vector Labs, United States) at 1 μg/ml, both prepared in PBS. WGA-AF488 selectively binds to *N*-acetylglucosamine and *N*-acetylneuraminic acid (sialic acid) residues highlighting the cell membrane of cardiomyocytes. DAPI was used as a cell nuclei marker. After staining, slides were washed twice during 2 min with PBS and mounted using Fluorescence Mounting Medium (Dako, United States). Images were acquired with 40X magnification in confocal microscope Nikon A1R+ (Japan) and the cross-sectional area of cardiomyocytes was quantified with NIS-Elements software (Nikon, Japan). Cardiomyocytes were selected for analysis only when their nuclei were seen in the center and their sarcolemmal membranes were well identified. The cross-sectional area of >200 cardiomyocytes was measured.

### Cardiomyocytes Isolation and [Ca^2+^]_i_ Study

**FIGURE 1:**
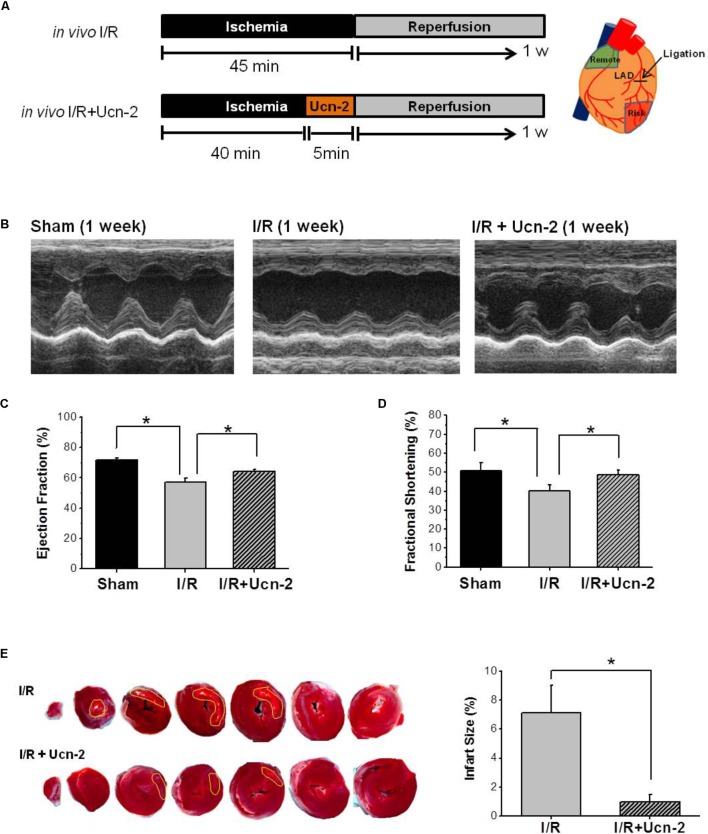
Urocortin-2 recovers cardiac contractility and reduces infarct size 1 week after I/R. **(A)** Experimental protocol for *in vivo* I/R animal model infused or not with Ucn-2. Tissue was isolated from remote (green area) and risk (red area) as drawn in right. **(B)** Representative M-mode echocardiographic images evaluated 1 week after surgery. Images are from “Sham” (n = 8); “I/R” rats (*n* = 15); and I/R rats infused with Ucn-2 (150 μg/Kg) before reperfusion (*n* = 14). **(C,D)** Bar graphs summarize Ejection Fraction (EF, %) and Shortening Fraction (SF, %) measured in same experimental groups as in **(B)**. Black bar is for “Sham”; gray bar is for “I/R”; and hatched gray bar is from “I/R + Ucn-2”. **(E)** Representative TTC stained transverse heart sections from I/R ± Ucn-2 rats. White area indicates necrosis. Bar graph in right shows the average of the infarct size (%). Values are mean ± SEM. ^∗^Indicates significance at *p* < 0.05.

Ventricular myocytes were isolated using collagenase type II (251 IU/mL; Worthington Biochemical, Lakewood, NJ, United States) as described previously (Domínguez-Rodríguez et al., 2015). The hearts were removed and perfused on a Langendorff perfusion apparatus. After perfusion, hearts were left in Petri dishes containing enzyme solution supplemented with 2 g/L Bovine Serum Albumin (BSA) and cut up into two regions of interest: the (non-infarcted) remote zone and (infarcted) risk zone as previously described (Driesen et al., 2007; van Rooij et al., 2008). As shown in **Figure 1A**, the areas below and adjacent of the LAD ligature were considered risk areas (highlighted by red color), whereas the non-infarcted remote zones were considered areas above the LAD ligature as indicated by the green color in **Figure 1A**. Both zones were gently stirred for 2–3 min at 37°C to disperse. Isolated cells were then filtered, centrifuged and suspended in Tyrode solution containing (in mM): 130 NaCl, 1 CaCl_2_, 0.5 MgCl_2_, 5.4 KCl, 22 glucose, 25 HEPES, 0.4 NaH_2_PO_4_, 5 NaHCO_3_; pH was adjusted to 7.4 with NaOH. Cells were plated in control solution containing 1.8 mM CaCl_2_ at 37°C and experiments were performed on Ca^2+^-tolerant rod-shaped myocytes at 37°C.

Isolated ventricular myocytes were loaded with Fluo-3 AM (6 μM) for 30 min, as described previously (Domínguez-Rodríguez et al., 2015). Only rod-shaped cells, quiescent when unstimulated were used for the Ca^2+^ experiments performed at room temperature (24–26°C) and under control Tyrode perfusion. Confocal Ca^2+^ images were obtained with confocal microscope (Leica SP5, objective w.i. 63x) by exciting cells at 500 nm and emission was collected at >510 nm using a white light laser in the line-scan mode. To record transients of [Ca^2+^]_i_, Fluo-3 loaded myocytes were excited at 1 Hz by electrically field stimulated using two parallel Pt electrodes until steady state before recording. To obtain F/F_0_ fluorescence’s values (F) were normalized by the basal fluorescence (F_0_). For SR Ca^2+^ load estimation, intact cardiac myocytes were rapidly perfused with 10 mM caffeine right after field-stimulation in order to empty SR. The amplitude of caffeine-evoked intracellular Ca^2+^ transient (F/F_0_) was used to assess SR Ca^2+^ load. Data were analyzed by IDL software (Exelis Visual Information Solutions, United States).

### Neonatal Rat Ventricular Myocytes Primary Culture

Neonatal rat ventricular myocytes were isolated from hearts of 1- to 3-days-old Wistar rats (Sabourin et al., 2016). The auricles were discarded and ventricular cells were dispersed by successive enzymatic digestion with 0.125% trypsin-DNAase (Sigma-Aldrich, United States). NRVMs (1 × 10^6^/ml) were seeded into plates. Primary ventricular cardiomyocytes were cultured in Dulbecco’s Modified Eagle Medium DMEM/medium 199 (4:1) supplemented with 10% horse serum, 15% fetal bovine serum (FBS, Thermo Fisher Scientific, United States), 1% glutamine, 100 U/ml penicillin and 100 μg/ml streptomycin for 24 h. On the next day, the medium was changed. Approximately 48 h after isolation, cells displayed as confluent monolayer with spontaneous contractile activity. Then, isolated cells were cultured in medium until their use.

### NRVMs Transfections and Urocortin-2 Treatment

Neonatal rat ventricular myocytes were transfected with siRNA using Lipofectamine^®^ RNAiMAX Transfection Reagent (Thermo Fisher Scientific, United States) when they were 70–80% of confluence according to the manufacturer’s instructions. Briefly, we diluted 5 μl of Lipofectamine RNAiMAX Reagent in 150 μl of Opti-MEN^®^ Medium (Gibco). Then, we added 3 μl at 10 μM of siRNA of TRPC5 (Dharmacon, United States) and Orai1 (Ambion, Thermo Fisher, United States). Preparations were mixed in 1:1 proportion and incubated for 5 min at room temperature. Finally, we added siRNA-lipid complex to cell culture. Cells were kept in culture for further 48–72 h and BLOCK-iT^TM^ Alexa Fluor^®^ Red MEM^®^ Medium was replaced each 24 h. BLOCK-iT^TM^ Alexa Fluor^®^ Red (Thermo Fisher Scientific, United States) was added as positive control to visualize the efficiency of cells transfection.

Neonatal rat ventricular myocytes were incubated in a simulated ischemic solution (mM): 142 NaCl, 3.6 KCl, 1.2 MgCl_2_, 1.8 CaCl_2_, 5 NaHCO_3_, 20 Hepes, 20 Lactate-Na, 20 sucrose (pH 6.22), then placed in an incubator of hypoxia at 1% O_2_ and 5% CO_2_. Reperfusion was restored by the removal of the ischemic solution and cells incubation an incubator at 21% O_2_ and 5% CO_2_.

We considered the following experimental groups: Group 1 (Control): Untreated NRVMs. Group 2 (I/R): After stabilization in control solution, NRVMs were exposed to simulated ischemia solution during 3 h and reperfused for 72 h with freshly control solution. Group 3 (Ucn-2): Same as group 2, but Ucn-2 (10 nM) was added during ischemia. Group 4 (Ucn-2 + Ast): Same as group 3, but Ucn-2 was added in presence of astressin (500 nM), CRF-R2 specific inhibitor.

### *In Situ* Proximity Ligation Assay

Spatial co-localization of TRPC5 and Orai1 were analyzed with PLA technique in NRVMs cell culture. The method used the Duolink *in situ* PLA detection kit Red (Sigma-Aldrich, United States) as previously described (Söderberg et al., 2008). NRVMs were seeded in a six-channel plate and fixed with 100% cold methanol during 5 min. After blocking for 30 min with 1% BSA and 3% heat-inactivated goat serum in PBS, cells were incubated with primary antibodies (rabbit anti-TRPC5, 1:50 (Dharmacon, United States) and mouse anti-Orai1, 1:100 (Novus Biologicals, United States) for 2 h at room temperature. Then, the probes Duolink PLA anti-rabbit PLUS and anti-mouse MINUS were added for 1 h at 37°C. The secondary antibodies of these probes were attached to synthetic oligonucleotides, which hybridize when they are in close proximity (i.e., 40 nm separation). The hybridized oligonucleotides were ligated for 30 min at 37°C and then they were amplified for 100 min at 37°C. The result red fluorescence is from the labeled oligonucleotides hybridized to the rolling circle amplification product and it was visualized using a confocal microscope (Leica TCS SP2, Germany). Maximum intensity projections of all z-sections (0.4 μm) were obtained by ImageJ software and puncta of maximum intensity projections were analyzed by Duolink ImageTool software (Sigma-Aldrich, United States). As a positive control we used the interaction between anti-MHC and anti-vimentin antibodies. The negative control was obtained using TRPC5 antigen together with anti-Orai-1 antibody, which did not show any detectable PLA signal.

### RNA Extraction and qRT-PCR Analysis

Rat’s hearts were washed in PBS, and immediately separated from risk and remote zones. Each zone was cut in small pieces collected in RNA later (Qiagen N.V., Germany), and processed according to the extraction of RNA. Total RNA was extracted from tissues by homogenization using tissuelyzer (Qiagen N.V., Germany) with 1 ml of Trizol. After chloroform mix and centrifugation, aqueous phase was transferred to “RNeasy mini kit” (Qiagen N.V., Germany) columns and were continued according to the manufacturer’s instructions. The ratio of absorbance at 260 and 280 nm (A260/A280) was used to assess the purity of RNA. RNA concentrations were determined from absorbance at 260 nm (A260). Two microgram of RNA were retrotranscribed to cDNA with the “RT quantitec kit” (Qiagen N.V., Germany). Standard qRT-PCRs were performed as described previously (Díaz et al., 2017). Data analysis was made with the “Expression Suite” software and fold change quantification was calculated using the comparative cycle threshold CT (ΔΔCT) method. 18s rRNA gene was used as endogenous control.

### Gene Expression Assay by Array Card

qRT-PCR was performed with the use of a Viia7 Real-Time PCR system (Applied Biosystems, United States) and Custom TaqMan^®^ Array cards (384 wells) was designed to detect expression of 45 genes of interest according to manufacturer’s indication (Applied Biosystems, United States). PCR mix was performed in a total volume of 125 μl, which included 2x Taqman PCR master mix and 1 μg of RNA. Thermal cycling conditions were as follows: 95°C for 5 min, followed by 40 cycles of 95°C for 10 s, and 60°C for 30 s.

### Western Blotting

Rat’s hearts were isolated, washed in PBS, and immediately separated from risk and remote zones. Small pieces of heart were disrupted through high-speed shaking in plastic tubes with stainless steel in the homogenizer tissuelyzer (Qiagen N.V., Germany). Protein samples were also processed from NRVMs culture. Then protein extraction was carried out with 1 ml of NP40 cell lysis buffer supplemented with inhibitor cocktail (Roche, Switzerland) and 1% of PMSF and incubated for 30 min on ice. After centrifugation, protein fractions were separated and quantified by Bradford method. Similar amounts of protein samples were subjected to SDS-PAGE (10% acrylamide) and electrotransferred onto PVDF membranes. After blocking with 5% non-fat dry milk dissolved in Tris-buffered saline containing 0.1% Tween-20 (TTBS) for 1 h at 37°C, membranes were probed overnight at 4°C with specific primary antibodies in TTBS with 1% of BSA. After washing, membranes were incubated for 45 min at room temperature with a horseradish peroxidase conjugated anti IgG (Cell Signaling, United States). Detection was performed with the enhanced chemiluminescence reagent ECL-prime (Amersham Bioscience, United Kingdom) in the ImageQuant LAS 4000 mini (GE Healthcare, United States). For quantification, the images were analyzed with ImageJ software using GAPDH or β-actin as housekeeping control. Antibodies: TRPC1, TRPC4, TRPC5 and TRPC6 (Alomone Labs, Israel), Orai1 (Novus Biologicals, United States), STIM1 (Sigma-Aldrich, United States) and GAPDH (Cell Signaling, United States).

### Statistical Analysis

Analyses were made with Origin 8.0 software. A sample size calculation was performed prior the start of this study. Values are reported as mean ± SEM. Difference between two groups was assessed with two-tailed unpaired Student’s *t*-test and between at least three groups with one-way or two-way ANOVA for multiple comparisons. The statistical significance was defined by a value of: ^∗^*p* < 0.05, ^∗∗^*p* < 0.01, ^∗∗∗^*p* < 0.001.

## Results

### Urocortin-2 Improves Heart’s Performances, Decreases the Infarct Size and Attenuates Fibrosis

The effect of Ucn-2 (150 μg/kg) infused 5 min before the opening of LCA was evaluated *in vivo* in rat model of I/R as indicated in **Figure 1A**. **Figures 1B–D** show significant reduction in the contractile capacity of the heart 1 week after I/R as assessed by M-Mode echocardiography. As depicted in **Figures 1C,D**, significant decrease in the Ejection Fraction (EF = 56.9% ± 3.3) and the Fractional Shortening (FS = 40.1% ± 3.4) was observed in I/R group compared to Sham (EF = 71.8% ± 1.3 and FS = 50.7% ± 4.0). Meanwhile, the infusion of rats with Ucn-2 before reperfusion recovered significantly EF (64.12% ± 1.53), FS (47.6% ± 0.9) and other hemodynamics parameters as summarized in **Table 1**. Interestingly, the beneficial effects of a single infusion of Ucn-2 on the ejection fraction and fractional shortening was observed as early as 24 h after the intervention and was even maintained 4 weeks after surgery (data not shown). Furthermore, rats’ infusion with Ucn-2 decreased significantly the infarct size associated with transient LCA ligation as illustrated in **Figure 1E**.

**Table 1:** Echocardiographic parameters of rat’s model of I/R.

Parameter	HR (bum)	EF (%)	FS (%)	LVESD (mm)	LVEDD (mm)	SV (μL)	DV (μL)	*n*
Sham	436 ± 5.3	71.8 ± 1.2	50.7 ± 4.0	2.7 ± 0.2	6.4 ± 0.1	119.5 ± 5.5	427.4 ± 19.9	8
I/R	424.2 ± 6.3	54.5 ± 2.8*	40.1 ± 3.4*	4.1 ± 0.3**	6.8 ± 0.2*	207.8 ± 24.01*	494.5 ± 36.4	15
I/R + Ucn-2	416.3 ± 3.8	65.2 ± 1.1*	47.6 ± 0.9*	3.5 ± 0.1*	5.9 ± 0.1	142.7 ± 7.8 **|	397.4 ± 13.8 **	16


*Data summary (mean ± SEM) of rats’ hemodynamic parameters evaluated in “Sham,” “I/R” and “I/R + Ucn-2” experimental groups after 1 week I/R intervention. HR, Heart Rate; EF, Ejection Fraction; FS, Fractional Shortening; LVESD, Left-Ventricle End-Systolic Diameter; LVEDD, Left-Ventricle End-Diastolic Diameter; SV, Systolic Volume; DV, Diastolic Volume. Data are mean ± SEM. ^∗^ and ^∗∗^ Indicate significance at *p* < 0.05 and *p* < 0.01 respectively. IR was compared with Sham and I/R+Ucn-2 vs. I/R.*

**FIGURE 2:**
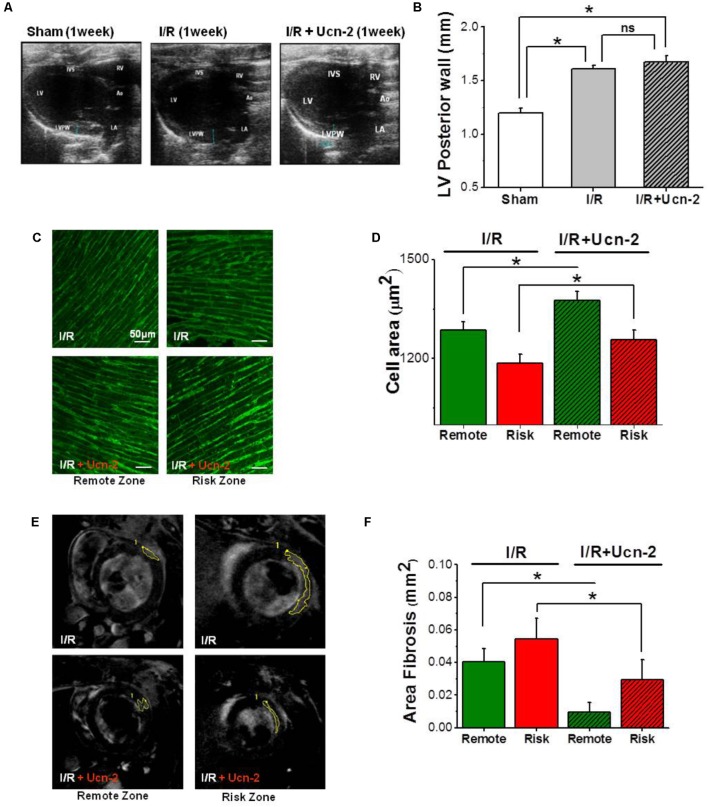
Urocortin-2 preserves cardiac hypertrophy, but it prevents fibrosis. **(A,B)** Representative and summary data of two-dimensional echocardiographic images in parasternal long-axis view of left ventricle taken from “Sham” (white bar, *n* = 8), “I/R” (gray bar, *n* = 15) and “I/R + Ucn-2” (hatched gray bar, *n* = 10) groups. **(C,D)** Representative images and summary data of staining with Wheat Germ Agglutinin (WGA) of heart section taken from risk (red) and remote (green) zones from I/R’s group (*n* = 6). Hatched bars are for I/R + Ucn-2 group. **(E)** *In vivo* cardiovascular magnetic resonance showing original images with mark of gadolinium enhancement area in remote and risk zones taken from I/R (up) and from I/R + Ucn-2 (down). **(F)** Bar graph summarizing fibrosis area (mm^2^) in remote (green) and risk (red) zones. LV, Left Ventricle; RV, Right Ventricle; LVPW, Left Ventricle Posterior Wall; IVS, Interventricular Septum; Ao, Aorta; LA, Left Atrium. Data are presented as mean ± SEM. ^∗^Indicates significance at *p* < 0.05.

Next, we examined I/R induced hypertrophy using echocardiography and WGA staining. **Figures 2A,B** show a significant increase in the thickness of left ventricle posterior wall (LVPW) in rat from I/R group, but also in rats infused with Ucn-2 (**Figures 2A,B**). The staining of heart’s section with WGA confirms a significant increase of cell area in remote and risk zones, both in I/R rats and in rats treated with Ucn-2 as shown in **Figures 2C,D**.

Afterward, we evaluated fibrosis *in vivo* 1 week after I/R using cardiovascular magnetic resonance performed with delayed enhancement following the administration of gadolinium. **Figures 2E,F** show the appearance of significant reactive fibrosis both in risk and remote zones as early as 1 week after surgery. Meanwhile, rats’ infusion with Ucn-2 efficiently prevented this fibrosis in both areas.

Altogether, these data indicate that the administration of Ucn-2 in reperfusion recovers significantly cardiac hemodynamic functions, reduces infarct size, attenuates fibrosis but it doesn’t prevent cardiac hypertrophy.

### Urocortin-2 Prevents I/R Dysregulation of [Ca^2+^]_i_ Handling in Cardiomyocytes

**FIGURE 3:**
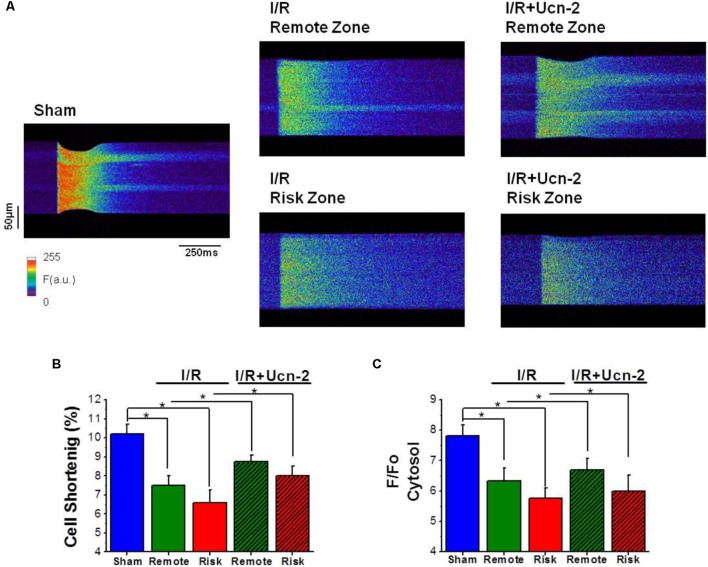
Urocortin-2 recovers the amplitude of [Ca^2+^] transients and the contraction of cardiomyocytes decreased under I/R. **(A)** Line-scan images during field stimulation at 1 Hz of cardiomyocyte isolated from “Sham” rats and from remote and risk zones of rat exposed to I/R infused or not with Ucn-2 (150 μg/Kg) 5 min before reperfusion. **(B)** Bar graph showing cellular shortening expressed in percentage of cell length in each experimental group. “Sham,” blue bar; “I/R” remote, green bar; and risk, red bar. Hatched bars are for “I/R + Ucn-2.” **(C)** Average of the amplitude of [Ca^2+^]_i_ transients measured as peak of F/F_0_, where F is the fluorescence and F_0_ is the fluorescence in the diastolic period. Data are from 6 “Sham” rats (*n* = 51 cells), 7 “I/R” rats (*n* = 32–42 cells) and 6 rats of “I/R + Ucn-2” group (*n* = 18–20 cells). Data are presented as mean ± SEM. ^∗^Indicates significance at *p* < 0.05.

Since Ca^2+^ homeostasis is crucial for heart contractility, we examined [Ca^2+^]_i_ handling in cardiomyocytes isolated from I/R rats after Ucn-2 infusion. **Figure 3A** shows representative line-scan Ca^2+^ fluorescence images obtained after electric field stimulation at 1 Hz. As indicated, rats undergoing I/R showed significant decrease in cellular contractions measured as cell shortening (**Figure 3B**) and in the amplitude of [Ca^2+^]_i_ transients recorded in isolated cardiac myocytes from remote and risk zones (**Figure 3C**), compared to Sham. SR Ca^2+^ load and the decay time, analyzed by caffeine-evoked [Ca^2+^]_i_ transients, were also significantly affected by I/R, being more prominent in risk than in remote zone as shown in Supplementary Figures 1A–C. Interestingly, the administration of Ucn-2 to I/R rats recovered significantly cell shortening and the amplitude of [Ca^2+^]_i_ transients as illustrated in **Figures 3A–C**, confirming that Ucn-2 is able to modulate of [Ca^2+^]_i_ when administrated in the onset of reperfusion.

### Urocortin-2 Regulates the Expression of Proteins Involved in Ca^2+^ Homeostasis

**FIGURE 4:**
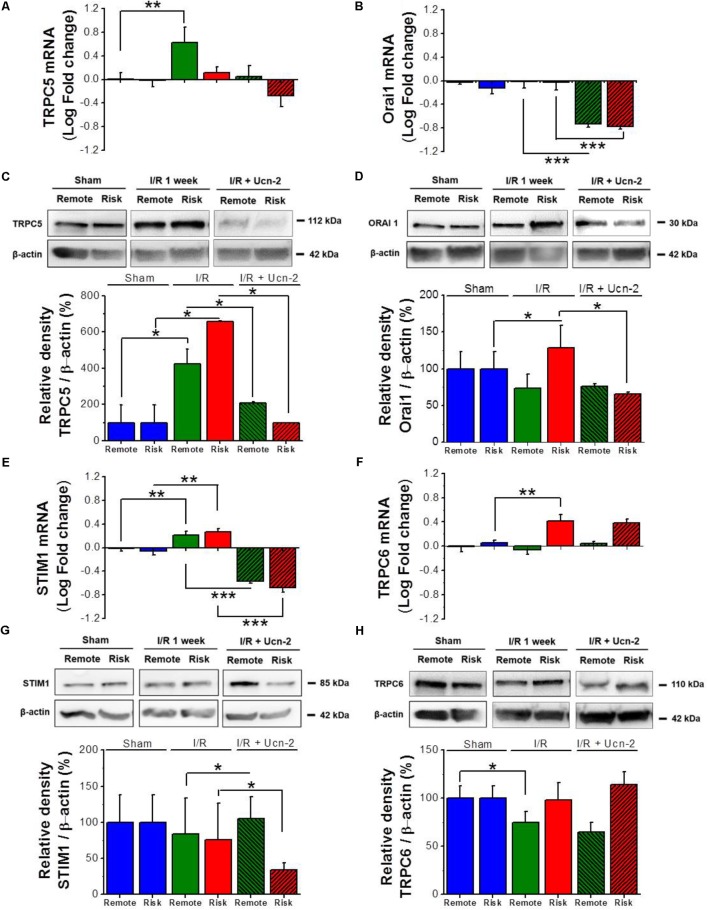
Urocortin-2 regulates the expression of TRPC5, Orai1 and STIM1. **(A,B,E,F)** Bar graphs showing relative fold change of mRNA levels of TRPC5, Orai1, STIM1 and TRPC6 respectively. **(C,D,G,H)** Representative immunoblots (top) and quantification (bottom) of protein expression of TRPC5, Orai1, STIM1 and TRPC6 respectively assessed by Western Blots and normalized to their corresponding β-actin expression. Sample were from “Sham” (blue bar); remote (green) and risk (red) zones processed 1 week after I/R. Hatched bars are for rats infused with Ucn-2 (150 μg/Kg). Values are mean ± SEM from 4 to 6 rats. ^∗^, ^∗∗^, ^∗∗∗^ Indicate significance at *p* < 0.05, *p* < 0.01, and *p* < 0.001 respectively.

In order to determine the molecular participants involved in I/R induced alteration of [Ca^2+^]_i_ homeostasis, we performed a PCR-based micro-array to evaluate the expression of 45 genes related to Ca^2+^ homeostasis in risk and remote zones. **Table 2** shows that 14 of the examined genes were significantly upregulated either in risk or in remote zones as compared to Sham. Interestingly, most of the upregulated genes belong to TRPC family (TRPC1/3/5/6) and to the store operated Ca^2+^ signaling pathway (Orai1/2 and STIM1/2). Therefore, we sought to validate the expression of these genes and we explored their possible regulation by the infusion of Ucn-2 in rat’s model of I/R. **Figures 4A,C** confirm that both mRNA and protein expression of TRPC5 were increased significantly in I/R rats in remote and/or risk zones. Similarly, the expression of Orai1 was increased especially in risk zone at the protein level (**Figures 4B,D**). Importantly, I/R rats’ infusion with Ucn-2 efficiently decreased the overexpression of TRPC5 and Orai1 in both zones. Ucn-2 treatment also downregulated the expression of STIM1 particularly in risk zone at mRNA and protein levels, meanwhile it didn’t affect the expression of TRPC6 (**Figures 4E–H**). In contrast, Ucn-2 failed to prevent I/R-evoked upregulation of other proteins such as Epac and NCX observed especially in risk zones (Supplementary Figure 2).

**Table 2:** List of the expression of genes dysregulated after I/R.

	Risk zone		Remote zone	
		
Gene	Fold change	*p*-value	Fold change	*p-value*
*Serca*	1.28 ± 0.13	0.179	**2.12 ± 0.3**	**0.001**
*Cacna1c*	**1.39 ± 0.18**	**0.079**	**2.07 ± 0.34**	**0.001**
*Orai1*	**1.45 ± 0.11**	**0.002**	**1.21 ± 0.08**	**0.075**
*Rapgef3*	**1.47 ± 0.16**	**0.002**	**1.28 ± 0.09**	**0.022**
*Trpc1*	**1.57 ± 0.11**	**0.002**	**1.61 ± 0.16**	**0.002**
*Hcn4*	**1.71 ± 0.36**	**0.033**	**2.76 ± 0.48**	**0.001**
*Trpc5*	1.71 ± 0.56	0.172	**10.87 ± 7.49**	**0.020**
*Trpc3*	**1.78 ± 0.21**	**0.001**	**1.42 ± 0.23**	**0.034**
*Slc8a1*	**1.83 ± 0.18**	**0.001**	**2.27 ± 0.23**	**0.001**
*Trpc4*	**2.08 ± 0.2**	**0.008**	**1.83 ± 0.19**	**0.041**
*Stim1*	**2.15 ± 0.27**	**0.001**	**1.72 ± 0.23**	**0.003**
*Stim2*	**2.38 ± 0.3**	**0.001**	**1.56 ± 0.12**	**0.001**
*Trpc6*	**3.89 ± 0.88**	**0.001**	1.03 ± 0.16	0.381
*Orai2*	**4.07 ± 1.35**	**0.001**	1.22 ± 0.24	0.197


*The PCR-based array was conducted in heart’s samples taken from “Sham,” and from risk and remote zones of “I/R” rats model. Data are expressed in fold change and in mean ± SEM (*n* = 4). Bold letters are for genes with significant changes at *p* < 0.05. Genes name: *Cacna1c*, Calcium voltage-gated channel subunit alpha1 C; *Hcn4*, Hyperpolarization activated cyclic nucleotide gated potassium channel 4 (I_*funny*_); *Orai1/2*, ORAI calcium release-activated calcium modulator 1/2; *Rapgef3*, Rap guanine nucleotide exchange factor 3 (Epac); *Serca*, Sarco/endoplasmic reticulum Ca^2+^-ATPase; *Slc8a1*, Solute carrier family 8 (sodium/calcium exchanger) member 1; *Stim1/2*, Stromal interaction molecule 1/2; *Trpc1/3/5/6*, Transient receptor potential cation channel subfamily C member 1/3/5/6.*

These data indicate that Ucn-2 is able to prevent I/R-evoked alteration of the expression of several proteins involved in the regulation of [Ca^2+^]_i_ homeostasis, which might play a significant role in heart contraction.

### Urocortin-2 Decreases Store-Operated Ca^2+^ Entry Induced by I/R in NRVMs

**FIGURE 5:**
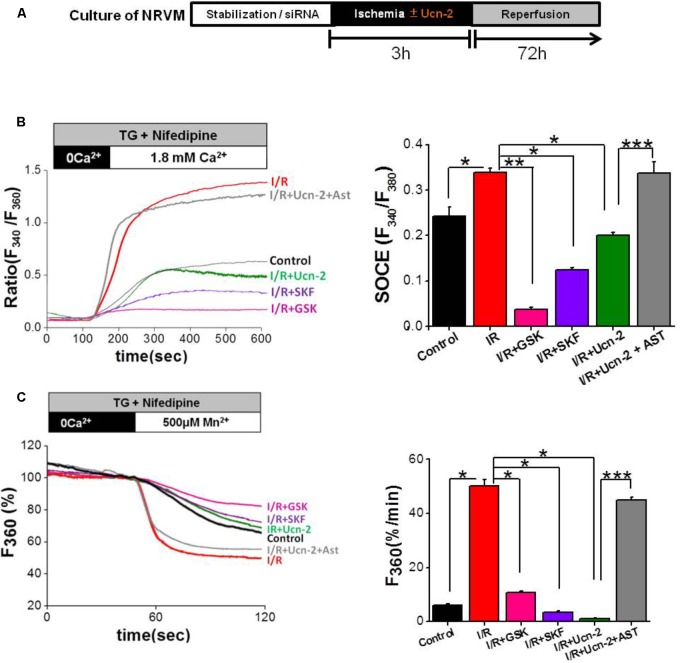
Urocortin-2 inhibits I/R-induced exacerbated SOCE in NRVMs. **(A)** Experimental protocol of cultured NRVMs subjected to I/R and Ucn-2 (10 nM). **(B)** Representative traces (left) showing the changes in [Ca^2+^]_i_ in Fura-2 loaded NRVMs, presented as ratio (F_340_/F_380_). In right, bar graph illustrating data summary of experiments as in left. **(C)** Representative traces (left) and data summary (right) of Mn^2+^ influx-induced Fura-2 quenching expressed in percent change of F_360_ fluorescence after the administration of Mn^2+^ (500 μM). Thapsigargin (TG, 2 μM) and nifedipine (NIF, 10 μM) were applied 4 min in the absence of extracellular Ca^2+^ and then Ca^2+^ (1.8 mM) was added as indicated. “Control” indicates responses of NRVMs to TG, “*I/R*” is for NRVMs under protocol of I/R, “*I/R+Ucn-2*” is NRVMs treated with Ucn-2 during I/R, “*I/R+Ucn-2+Ast*” indicates NRVMs pre-incubated with astressin (0.5 μM) before Ucn-2 addition in I/R, “*I/R+SKF*” indicates NRVMs pre-incubated with SKF-96365 (40 μM), and “*I/R + GSK*” indicates NRVMs pre-incubated with GSK-7975A (10 μM). *n* = 100–250 cells from 5–10 cultures. Data are mean ± SEM. ^∗^, ^∗∗^, ^∗∗∗^ indicate significance at *p* < 0.05, *p* < 0.01, and *p* < 0.001 respectively.

Taking into consideration the previous data demonstrating that I/R promotes the alteration of several cationic channels related to SOCC pathway, we examined whether I/R promotes SOCE and if Ucn-2 regulates SOCE. We used a classical protocol for SOCC activation in NRVMs under I/R *in vitro* as illustrated in **Figure 5A**. **Figure 5B** shows that the addition of thapsigargin (TG, 2 μM), in presence of nifedipine (NIF, 10 μM), to control NRVMs evoked significant increase in the [Ca^2+^]_i_ after Ca^2+^ restoration. Importantly, when NRVMs were subjected to I/R TG-induced larger augmentation of [Ca^2+^]_i_ amplitudes compared to control, which was significantly blunted in cells pre-treated with SOCC inhibitors GSK-7975A (10 μM) and SKF-96365 (40 μM). Furthermore, we measured Mn^2+^-induced quenching of Fura-2 fluorescence in NRVMs treated with TG to obtain direct evidences that I/R promoted exacerbated SOCE due to SOCC activity. **Figure 5C** shows that TG (2 μM) promoted significant Mn^2+^ influx as observed by fast decay of Fura-2 fluorescence after Mn^2+^ (500 μM) addition in NRVMs submitted to I/R compared to control. The observed Mn^2+^ influx was significantly reduced by GSK-7975A and SKF-96365 confirming SOCE stimulation.

Interestingly, the addition of Ucn-2 (10 nM) to NRVMs prevented the augmented TG-induced Ca^2+^ and Mn^2+^ influx under I/R (**Figures 5B,C**). Besides, NRVMs pre-treatment with astressin (1 μM), inhibitor of CRF-R2, abolished the effect of Ucn-2 on Ca^2+^ and Mn^2+^ influx, confirming that Ucn-2 acts through its receptor CRF-R2 to inhibit SOCE exacerbated by I/R. These results demonstrate that I/R potentiates SOCE, which is inhibited by Ucn-2.

### Urocortin-2 Attenuated -SOCE Is Dependent on TRPC5 and Orai1 Channels

**FIGURE 6:**
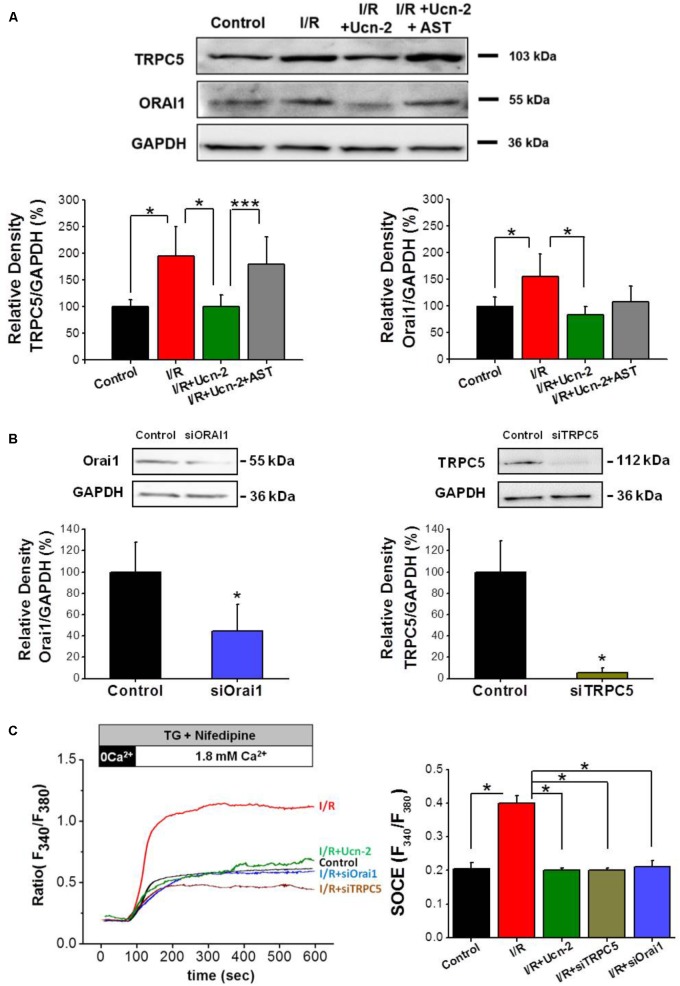
Urocortin-2 inhibition of I/R-induced SOCE involves TRPC5 and Orai1 activation. **(A)** Representative Western Blot (top) and summary analysis (bottom) showing the expression of TRPC5 and Orai1 normalized to GAPDH examined in untreated NRVMs (control); after I/R, and in cells treated with Ucn-2 ± 0.5 μM of astressin (*I/R + Ucn-2; I/R + Ucn-2* + *Ast).* **(B)** Representative Western Blot (top) and summary analysis (bottom) showing the expression of TRPC5 and Orai1 normalized to GAPDH examined in untreated NRVMs (control); and in cells transfected with siRNA against Orai1 (siOrai1) and TRPC5 (siTRPC5). **(C)** Representative traces and average data of TG-induced Ca^2+^ influx in Fura-2 loaded NRVMs from “Control,” “I/R” and “I/R+Ucn-2,” and in cells transfected with siRNA of TRPC5 “I/R+siTRPC5” and Orai1 “I/R+siOrai1.” *n* = 100–200 cells from 6 cultures. Data are mean ± SEM. ^∗^, ^∗∗^, ^∗∗∗^ indicate significance at *p* < 0.05, *p* < 0.01, and *p* < 0.001 respectively.

Based on the obtained data in adult cardiomyocytes, where we observed significant changes in the expression of TRPC5 and Orai1, we evaluated whether they are involved in SOCE induced by I/R in NRVMs. Firstly, **Figure 6A** shows that I/R increased the expression of TRPC5 and Orai1 in NRVMs. Importantly, cells treatment with Ucn-2 (10 nM) inhibited completely I/R-evoked TRPC5 and Orai1 overexpression. In contrast, cells pre-treatment with astressin (1 μM) blocked the effect of Ucn-2 on the expression of TRPC5 and Orai1. Secondly, we examined SOCE in NRVMs transfected with siRNA against TRPC5 and Orai1. **Figures 6B,C** shows that I/R-induced Ca^2+^ influx was completely inhibited when TRPC5 and Orai1 genes were silenced, confirming the implication of these proteins in I/R inducing exacerbated SOCE. Interestingly, the effect of TRPC5 and Orai1 downregulation was very similar to the inhibitory effect of Ucn-2 in SOCE.

**FIGURE 7:**
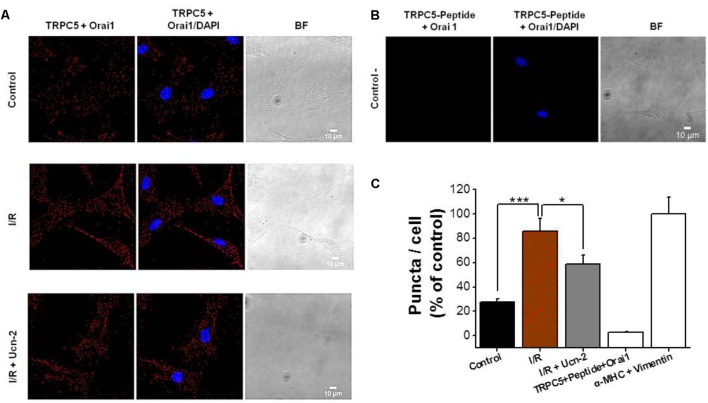
I/R enhances TRPC5 and Orai1 co-localization in cardiomyocytes. **(A)** Representative images of NRVMs using primary antibodies against TRPC5 and Orai1 conjugated with the appropriate Proximity Ligation Assay (PLA) probes, from untreated “control” cells, “I/R” cells and from cells incubated with Ucn-2 (10 nM; I/R + Ucn-2). The left panels show NRVMs images when antibodies were conjugated with PLA probes; middle panels show merged images with DAPI (blue); and right panels show Bright Field (BF) images. Red puncta indicate that proteins are in close proximity (<40 nm). **(B)** Images from NRVMs pre-incubated with control peptide antigen of TRPC5, anti-TRPC5 and with anti-Orai1 primary antibodies (negative control). **(C)** Bar graph summarizes the mean number of PLA signals in different experimental groups. *n* = 3 cell cultures. Data are presented as mean ± SEM normalized to positive control determined by the colocalization of vimentin and α-MHC (α Myosin heavy chain). ^∗^, and ^∗∗∗^ indicate significance at *p* < 0.05 and *p* < 0.001 respectively.

In light of the previous data, we examined the endogenous subcellular localization of TRPC5 and Orai1 and their possible interaction by the use of *in situ* PLA. **Figures 7A,C** show PLA red puncta in control NRVMs incubated with primary antibodies against TRPC5 and Orai1 indicating their close proximity. Furthermore, the number of PLA red puncta enhanced significantly under I/R, suggesting an increase of TRPC5 and Orai1 interaction. In contrast, NRVMs incubation with Ucn-2 during I/R significantly decreased puncta signals, indicating less interaction between both proteins. As a control, **Figures 7B,C** show that PLA signal decreased significantly in NRVMs pre-incubated with control peptide antigen of TRPC5 in presence of both TRPC5 and Orai1 antibodies. Taken together, these data suggest that TRPC5 interacts with Orai1 in basal conditions but they increase their association under I/R process, which certainly will promote Ca^2+^ entry and might be related to the abnormal [Ca^2+^]_i_ signaling in reperfused heart. These data shows that Ucn-2 efficiently inhibited SOCE through TRPC5 and Orai1 downregulation and interaction.

## Discussion

**FIGURE 8:**
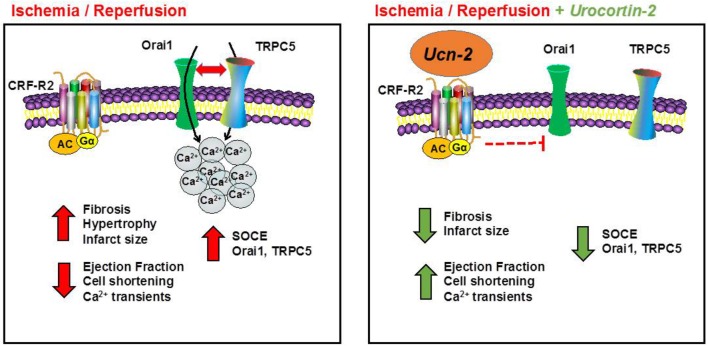
Schematic model illustrating the cardioprotection effect of Ucn-2 from I/R injuries. Left panel summarizes the effect of I/R in rats. I/R promotes fibrosis, hypertrophy and increases the infarct size. I/R also decreases heart’s contractility (ejection fraction and cell shortening) and [Ca^2+^]_i_ transients. In addition, we demonstrated that I/R potentiates SOCE and upregulated several ion channels, such as Orai1 and TRPC5. Right panel illustrates the effects of the addition of Ucn-2 at the onset of reperfusion. Ucn-2 inhibits fibrosis and decreases the infarct size; it recovers significantly heart’s contraction and the amplitude of [Ca^2+^]_i_ transients. Moreover, Ucn-2 blocks I/R-evoked exacerbated SOCE and it inhibits I/R-induced Orai1 and TRPC5 upregulation and interaction.

An effective and early coronary revascularization is considered the best therapy to limit the extent of myocardial infarction. Nevertheless, heart’s reperfusion is paradoxically associated with adverse effects that harm its adequate function (Hausenloy et al., 2017). Therefore, strategies of cardioprotection still remain of major interest to limit heart’s damage from I/R injury and the adverse ventricular remodeling (Heusch, 2015). Our study provides several new data describing that a single i.v. administration of Ucn-2, right before revascularization, protects efficiently the heart from I/R lesions. As summarized in **Figure 8**, Ucn-2 beneficial effects involve the recovery of cardiac contraction, the attenuation of fibrosis extension and the regulation of [Ca^2+^]_i_ signaling. We also provide the first quantitative description of Ucn-2 effects on I/R-activated SOCE in the heart, which correlated with the decrease in the expression and activity of TRPC5 and Orai1 channels either in risk or in remote zones.

We demonstrated that as soon as 1 week after I/R, significant structural and molecular alterations happen in risk zone, close to the infarcted area, but also in remote zone that is supposed to be healthy. Hypertrophy and fibrosis affected not only to the infarcted area but also the non-infarcted area, which is probably due to the initial multiphase reparative responses. Indeed, it is well known that the healing process triggered after a myocardial infarct is accomplished by a series of molecular reactions. These facts are: the release of cytokines, matrix metalloproteinases and growth factors; the recruitment of inflammatory cells; the differentiation of macrophages, mast cells and myofibroblasts, as well as the formation of new vessels and scar tissue (Talman and Ruskoaho, 2016; Montecucco et al., 2017). Consistent with our findings, these molecular reactions affect equally the damaged tissue in risk zone and the preserved tissue in remote zone, eliciting considerable heart adverse remodeling. Importantly, rats’ infusion with Ucn-2 prevented fibrosis in both areas in agreement with previous studies which showed that Ucn-2 decreased collagen deposition in the heart and suppressed the expression of fibrosis’ markers as TGF-β1 and collagen-1 (Rademaker et al., 2015). In contrast, Ucn-2 didn’t inhibit I/R-induced cardiac hypertrophy. Our observation agrees with others reports that described pro-hypertrophic effects of Ucn peptides in cultured NRVMs (Ikeda et al., 1998; Chanalaris et al., 2005). Indeed, isoforms of Ucn stimulated several markers of hypertrophy, increased cell size and promoted embryonic genes β-Myosin Heavy Chain (β-MHC) as well as Atrial and B-type Natriuretic Peptides (ANP and BNP). Nevertheless, other reports suggested an anti-hypertrophic action of chronic infusion of Ucn-2 in a rat model of arterial hypertension (Dieterle et al., 2009) and in mice model of heart infarct (Ellmers et al., 2015). Perhaps, Ucn-2 preserved the observed cardiac hypertrophy to maintain its compensatory effect at least in the early stage of heart remodeling as described previously (Rubin et al., 1983).

Cardiac dysfunction in reperfused hearts has been also related to cardiac [Ca^2+^]_i_ mishandling (Garcia-Dorado et al., 2012). Several mechanisms have been considered to explain the alteration of [Ca^2+^]_i_ during reperfusion and the most common changes associated with I/R are usually linked with acidosis, excitation-contraction coupling disruption, depression of SERCA activity and/or activation of NCX reverse mode (Inserte et al., 2002). In this way, we showed that several parameters of [Ca^2+^]_i_ homeostasis were significantly altered in risk zones, but unexpectedly also in remote zones. Furthermore, we determined significant changes in the expression of key proteins involved in SOCE pathway, such as Orai1, STIM1 and TRPC channels.

Compelling evidences demonstrated that TRPC and SOCC contributes to different cardiac pathology; nevertheless, their role in I/R is less explored (Smani et al., 2015; Eder, 2017). Here, we used a combination of functional Ca^2+^ imaging study, biochemical and immunostaining approaches to confirm that SOCE is observed and even potentiated by I/R in NRVMs. We demonstrated that widely used inhibitors of SOCC blocked TG-induced Ca^2+^ and Mn^2+^ influx, confirming SOCE activation under I/R. We specifically showed a pivotal role of Orai1 and TRPC5 as they were upregulated by I/R both in adult cardiac tissue and in NRVMs. We determined that I/R induced an exacerbated SOCE and an upregulation of TRPC5 and Orai1 which may objectively explain the significant increase of TRPC5 and Orai1 colocalization and interaction under I/R. It is well accepted that Orai1 is required for SOCE in neonatal and adult cardiomyocytes; however, the implication of TRPC5 in SOCE is less studied (Smani et al., 2015; Avila-Medina et al., 2018). Orai1 likely interacts with different TRPC to form a non-selective SOCC especially in excitable cells (Bush et al., 2006; Liao et al., 2008). A recent study showed that aldosterone enhanced SOCE and increased the expression of STIM1, TRPC1, TRPC4, TRPC5 and Orai1 channels (Sabourin et al., 2016). Others showed that TRPC1 and TRPC4 activation are associated with a passive Ca^2+^ influx induced by angiotensin II or isoproterenol, and are related to maladaptive cardiac hypertrophy (Camacho Londoño et al., 2015). In our experimental conditions, we did not observe significant changes in the expression of TRPC1 and TRPC4 under I/R (Data not shown). However, we demonstrated that TRPC5 and Orai1 are required for SOCE since their gene’s silencing inhibited completely I/R-induced SOCE. We also showed for the first time that Orai1 and TRPC5 are distributed in close sub-membrane domains in cardiac myocytes. Furthermore, we showed that TRPC5 and Orai1 increased their colocalization under I/R, which might explain the observed exacerbated SOCE.

Interestingly, we showed that *i.v.* infusion or cells treatment with Ucn-2 inhibited I/R-induced upregulation of TRPC5 and Orai1 in cardiac tissue isolated from rats’ I/R model and in NRVMS subjected to I/R *in vitro*. Actually, in NRVMs Ucn-2 prevented I/R-induced upregulation of TRPC5 and Orai1, inhibited I/R-evoked exacerbated SOCE, and significantly decreased the interaction between TRPC5 and Orai1. These effects were dependent on CRF-R2 activation since its inhibition blocked efficiently Ucn-2 actions. Our data are consistent with previous reports that showed Ucn-1 inhibition of SOCE in coronary smooth muscle cells (Smani et al., 2007) and skeletal muscle (Reutenauer-Patte et al., 2012). In addition to TRPC5 involvement in SOCE, several reports demonstrated that TRPC5 is sensitive to reactive oxygen species (ROS) (Wong et al., 2010; Shimizu et al., 2014), what can be triggered under I/R. Therefore, Ucn-2 downregulation of TRPC5 might mitigate the effect of ROS on cardiac cells integrity under I/R.

Taken together, our findings highlight new unexpected aspects of Ucn-2 role in cardiac protection. Ucn-2 improves cardiac functions and remodeling; modulates [Ca^2+^]_i_; and inhibits TRPC5 and Orai1 dependent SOCE. These protective effects, together with others Ucn-2’s actions including vasorelaxation of human coronary artery (Smani et al., 2011), its positive inotropism (Smani et al., 2010) or its regulation of endocrine and renal effects (Rademaker et al., 2011), suggest that Ucn-2 is a promising and valuable therapeutic drug to mitigate cardiac dysfunction in post-STEMI patients. Actually, recent reports showed that Ucn-2 administration improved left ventricle contractility in patients with decompensated or chronic heart failure (Chan et al., 2013; Stirrat et al., 2016).

## Author Contributions

TS, AD-R, AG, and AO conceived and designed the experiments. AD-R, IM-G, ID, EdR-dP, EC-S, JA-M, and AH performed the experiments. AD-R, AG, J-PB, AH, JR, AC, AO, and TS analyzed and discussed the data. TS wrote the paper.

## Conflict of Interest Statement

The authors declare that the research was conducted in the absence of any commercial or financial relationships that could be construed as a potential conflict of interest.

## Abbreviations

[Ca^2+^]_i_: intracellular Ca^2+^ concentration
CRF: corticotropin-releasing factor
Epac: exchange protein directly activated by cAMP
I/R: ischemia and reperfusion
LCA: left coronary artery
NCX: Na^+^/Ca^2+^ exchanger
NRVM: Neonatal Rat Ventricular Myocytes
PLA: proximity ligation assay
SOCE: store-operated Ca^2+^ entry
SOCC: store operated Ca^2+^ channels
SR: sarcoplasmic reticulum
STEMI: ST-segment-elevation myocardial infarction
TRPC: transient receptor potential canonical
Ucn-1/2/3: Urocortin peptides
