# Supplementary figures and images for: Urocortin-2 Prevents Dysregulation of Ca2+ Homeostasis and Improves Early Cardiac Remodeling After Ischemia and Reperfusion
Source: Front Physiol. 2018 Jul 3;9:813. doi: 10.3389/fphys.2018.00813 (PMC6037857; doi:10.3389/fphys.2018.00813)

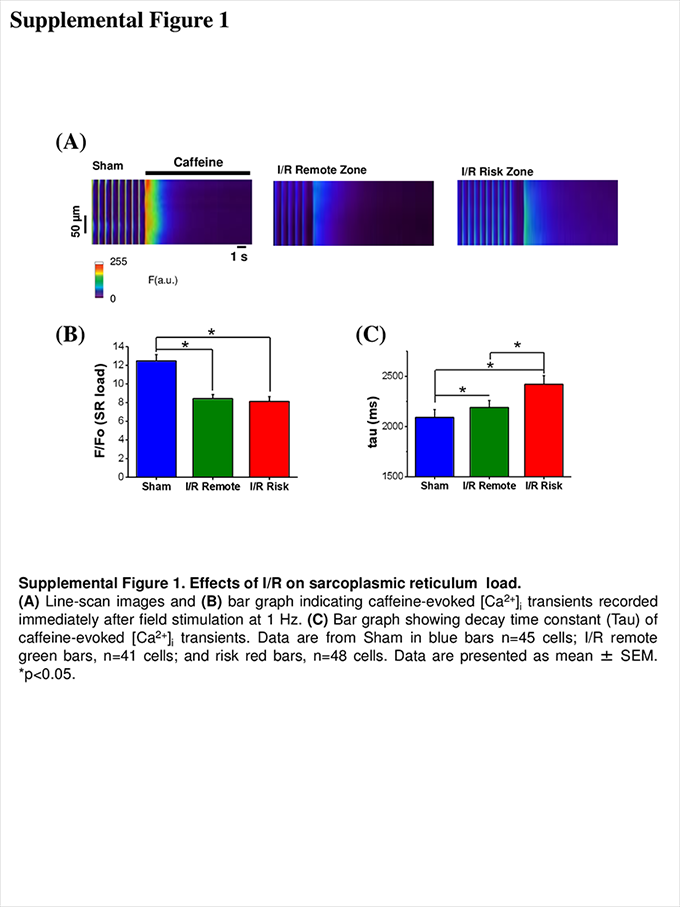

Supplement: Supplementary file 1 [file Image_1.tif]
